# Supplementary material for: Class B1 GPCR activation by an intracellular agonist
Source: Nature. 2023 Jun 7;618(7967):1085–93. doi: 10.1038/s41586-023-06169-3 (PMC10307627; doi:10.1038/s41586-023-06169-3)
Supplement: Supplementary file 1 — Reporting Summary [file 41586_2023_6169_MOESM1_ESM.pdf]

nature portfolio

Corresponding author(s): Osamu Nureki

Last updated by author(s): Mar 27, 2023

Reporting Summary

Nature Portfolio wishes to improve the reproducibility of the work that we publish. This form provides structure for consistency and transparency in reporting. For further information on Nature Portfolio policies, see our Editorial Policies and the Editorial Policy Checklist.

Statistics

For all statistical analyses, confirm that the following items are present in the figure legend, table legend, main text, or Methods section.

n/a	Confirmed	
☐	☒	The exact sample size (n) for each experimental group/condition, given as a discrete number and unit of measurement
☐	☒	A statement on whether measurements were taken from distinct samples or whether the same sample was measured repeatedly
☐	☒	The statistical test(s) used AND whether they are one- or two-sided<br>*Only common tests should be described solely by name; describe more complex techniques in the Methods section.*
☒	☐	A description of all covariates tested
☐	☒	A description of any assumptions or corrections, such as tests of normality and adjustment for multiple comparisons
☐	☒	A full description of the statistical parameters including central tendency (e.g. means) or other basic estimates (e.g. regression coefficient) AND variation (e.g. standard deviation) or associated estimates of uncertainty (e.g. confidence intervals)
☐	☒	For null hypothesis testing, the test statistic (e.g. F, t, r) with confidence intervals, effect sizes, degrees of freedom and P value noted<br>*Give P values as exact values whenever suitable.*
☒	☐	For Bayesian analysis, information on the choice of priors and Markov chain Monte Carlo settings
☒	☐	For hierarchical and complex designs, identification of the appropriate level for tests and full reporting of outcomes
☒	☐	Estimates of effect sizes (e.g. Cohen's d, Pearson's r), indicating how they were calculated

Our web collection on statistics for biologists contains articles on many of the points above.

Software and code

Policy information about availability of computer code

Data collection	Serial EM3.7.4, SoftMax Pro ver7.0.3, Zeiss ZEN 2.3 SP1 FP3 (black, 64 bit) (ver.14.0.21.201)
Data analysis	RELION-3.1, CTFFIND4.1, COOT-0.9.3 EL, PHENIX-1.14-3260, REFMAC5, CUEMOL-2.2.3.443, UCSF Chimera X 1.0, Prism 9, FIji (ver. 2.0.0-rc-69/1.52p), FlowJo, MODELLER 10.1, VMD 1.9.3, Charmm36, MemProtMD, CHARMM-GUI, NAMD 2.13, mdtraj-1.9.8

For manuscripts utilizing custom algorithms or software that are central to the research but not yet described in published literature, software must be made available to editors and reviewers. We strongly encourage code deposition in a community repository (e.g. GitHub). See the Nature Portfolio guidelines for submitting code & software for further information.

Data

Policy information about availability of data

All manuscripts must include a data availability statement. This statement should provide the following information, where applicable:

- Accession codes, unique identifiers, or web links for publicly available datasets
- A description of any restrictions on data availability
- For clinical datasets or third party data, please ensure that the statement adheres to our policy

Atomic coordinates for the PCO371-bound PTH1R–mini–Gsβ1γ2–Nb35 complex have been deposited in the Protein Data Bank under accession code 8GW8. The associated electron microscopy data have been deposited in the Electron Microscopy Data Bank under accession code EMD-34305. All other data provided with this paper.

Human research participants

Policy information about studies involving human research participants and Sex and Gender in Research.

Reporting on sex and gender	No concern about this section in this study.
Population characteristics	No concern about this section in this study.
Recruitment	No concern about this section in this study.
Ethics oversight	No concern about this section in this study.

Note that full information on the approval of the study protocol must also be provided in the manuscript.

Field-specific reporting

Please select the one below that is the best fit for your research. If you are not sure, read the appropriate sections before making your selection.

☒ Life sciences ☐ Behavioural & social sciences ☐ Ecological, evolutionary & environmental sciences

For a reference copy of the document with all sections, see nature.com/documents/nr-reporting-summary-flat.pdf

Life sciences study design

All studies must disclose on these points even when the disclosure is negative.

Sample size	Sample sizes were determined based on prior literature best practices in the field; no statistical methods were used to predetermine sample size. We performed three to five independent experiments and evaluated 21-26 cells in the cell-based functional assays and the confocal microscopy analysis, respectively, based on other similar methodologies (PMID 31160049, 35087057)
Data exclusions	No data was excluded from the analysis.
Replication	Cell-based experiments were independently performed at least three times. We verify that all experiments were successfully performed in duplicate.
Randomization	For cryo-EM studies, particles were randomly assigned to half-maps for resolution determination. Randomization was not relevant to the other experiments in our study as these assays don't have unknown covariates.
Blinding	Blinding was not relevant to the experiments in our study since no subjective allocation was involved in our study.

Reporting for specific materials, systems and methods

We require information from authors about some types of materials, experimental systems and methods used in many studies. Here, indicate whether each material, system or method listed is relevant to your study. If you are not sure if a list item applies to your research, read the appropriate section before selecting a response.

Materials & experimental systems

n/a	Involved in the study	
☐	☒	Antibodies
☐	☒	Eukaryotic cell lines
☒	☐	Palaeontology and archaeology
☒	☐	Animals and other organisms
☒	☐	Clinical data
☒	☐	Dual use research of concern

Methods

n/a	Involved in the study	
☒	☐	ChIP-seq
☐	☒	Flow cytometry
☒	☐	MRI-based neuroimaging

Antibodies

Antibodies used	anti-FLAG epitope tag (DYKDDDDK) mouse monoclonal antibody (Clone 1E6, FujiFilm Wako Pure Chemical, cat. no. 012-22384);<br>goat anti-mouse IgG secondary antibody conjugated with Alexa Fluor 488 (Thermo Fisher Scientific, cat. no. A32723);<br>anti-FLAG epitope tag (DYKDDDDK) mouse monoclonal antibody conjugated with Alexa Fluor 647(Clone FLA-1, MBL lifescience, cat. no. M185-A64)

Validation

All the commercial antibodies were verified by the manufactures according to immunoblots and images on their websites.
These were validated by their respective manufacturers as indicated at these links:
https://labchem-wako.fujifilm.com/jp/product/detail/W01W0101-2238.html
https://www.thermofisher.com/antibody/product/Goat-anti-Mouse-IgG-H-L-Highly-Cross-Adsorbed-Secondary-Antibody-Polyclonal/A32723
https://www.mblintl.com/products/m185-a64/

Eukaryotic cell lines

Policy information about cell lines and Sex and Gender in Research

Cell line source(s)

HEK293A purchased from Thermo Fisher Scientific (cat. no. R70507); HEK293S GnT1– purchased from American Type Culture Collection (cat. no. CRL-3022); E. Coli BL21 strain (DE3) purchased from New England Biolabs (cat. no. Cat# C2527), Sf9 (Gibco, cat. no. 11496015)

Authentication

None were authenticated.

Mycoplasma contamination

Not tested in Sf9; HEK 293A and HEK293S cells were regularly screened to ensure the absence of mycoplasma contamination using MycoAlert Mycoplasma detection kit (Lonza)

Commonly misidentified lines
(See ICLAC register)

None of commonly misidentified lines were used in this study.

Flow Cytometry

Plots

Confirm that:

☐ The axis labels state the marker and fluorochrome used (e.g. CD4-FITC).

☐ The axis scales are clearly visible. Include numbers along axes only for bottom left plot of group (a 'group' is an analysis of identical markers).

☐ All plots are contour plots with outliers or pseudocolor plots.

☐ A numerical value for number of cells or percentage (with statistics) is provided.

Methodology

Sample preparation

HEK293A cells were transfected by combining 3 µL of polyethylenimine solution (1mg mL-1) and 200 ng of a plasmid encoding FLAG epitope-tagged GPCR.

Instrument

EC800 flow cytometer (sony)

Software

FlowJo 10 (FlowJo), Prism 9 (GraphPad)

Cell population abundance

N/A

Gating strategy

Live cells were gated with a forward scatter (FS-Peak-Lin) cutoff of 390 setting a gain value of 1.7.

☐ Tick this box to confirm that a figure exemplifying the gating strategy is provided in the Supplementary Information.
